# Process Design and Parameters Interaction in Material Extrusion 3D Printing: A Review

**DOI:** 10.3390/polym15102280

**Published:** 2023-05-12

**Authors:** Ouri Bouzaglou, Ofek Golan, Noa Lachman

**Affiliations:** Department of Materials Science and Engineering, Tel Aviv University, Tel Aviv 6997801, Israel

**Keywords:** additive manufacturing, mechanical properties, polymers, printing parameters, governing mechanisms in FDM

## Abstract

Additive Manufacturing (AM), commonly known as “3D printing”, is rapidly integrated into many various fields, from everyday commercial to high-end medical and aerospace. Its production flexibility in small-scale and complex shapes is a significant advantage over conventional methods. However, inferior physical properties of parts manufactured by AM in general, and by material extrusion in particular, compared to traditional fabrication methods, inhibit its full assimilation. Specifically, the mechanical properties of printed parts are not high enough and, more importantly, not consistent enough. Optimization of the many various printing parameters is therefore required. This work reviews the influence of material selection, printing parameters such as path (e.g., layer thickness and raster angle), build (e.g., infill and building orientation) and temperature parameters (e.g., nozzle or platform temperature) on mechanical properties. Moreover, this work focuses on the interactions between the printing parameters, their mechanisms, and the statistical methods required to identify such interactions. Choosing the right parameters can increase mechanical properties by up to 60% (raster angle and orientation build), or render other parameters insignificant (material selection), while specific settings of certain parameters can completely inverse the influence trend of other parameters. Finally, trends for future research are suggested.

## 1. Introduction

Additive Manufacturing processes (AM) have been drawing increasing interest from industry as well as the research world and the academic community. It is a manufacturing technology that fabricates three-dimensional (3D) physical models directly from 3D Computer-aided design (CAD) data. The data is used to divide the design into thin layers in one direction, then a layered manufacturing process stacks and bonds these layers. In comparison with the previous numerically controlled manufacturing technology, AM can fabricate models with complex shapes without geometric restriction under controlled work conditions. Hence, this manufacturing technology has been widely applied in various fields, from industrial products to medical appliances. The most popular methods today are material extrusion (FDM/FFF), stereolithography (SLA) and selective laser sintering (SLS). According to Wohler’s Report, from 1995 to the present, over 11,300 rapid prototyping machine systems have been purchased and widely employed in new product developing processes. In regards to the materials used, polymers are the main raw material. Initially mainly limited to Acrylonitrile Butadiene Styrene (ABS) and Polylactic Acid (PLA), the range of plastics available for 3D printing, with variations of colors and properties, has grown considerably [1]. Thermoplastic materials and composites, originally reserved for prototyping purposes, are now advanced enough to allow additive manufacturing to be used to produce functional parts.

This research focuses on Fused Filament Fabrication (FFF) technology, which is one of the most widely used printing methods. FFF technology was invented in 1989 by Scott Crump, founder of Stratasys (which trademarked it under the name “Fused Deposition Modeling”, or FDM), which is now one of the largest 3D printer manufacturers. The FFF process is based on the well-known principle of material extrusion. In this process, the machine is fed with a thermoplastic polymer filament; the filament is pushed through a heated nozzle to produce a malleable wire of several micrometers in diameter. The 3D part is obtained by the continuous deposition of this thread, layer by layer, made by moving the nozzle, the tray of the printer, or both, in all directions of space [2].

FFF not only produces parts for use as prototypes, models and molds but also is increasingly used as a production technique for commercial products such as medical implants [3]. One of the biggest challenges in engineering FFF parts for end-use applications is predicting and assuring the mechanical properties of the FFF-made part [2]. Compared to conventional plastic processing techniques such as injection molding, FFF-made parts typically have lower mechanical properties due to the discontinuous nature of the process: adjacent fibers initially come into contact with one another while they are molten, but their rapid cooling process causes them to solidify prior to completely fusing with the other fibers, leaving voids between the fibers and interfaces with only partial cohesion [4].

The key aspects of functionality in FFF-made parts are surface roughness, dimensional accuracy and mechanical properties. In order to predict the behavior of the final printed parts, it is essential to understand the raw material properties in the FFF process and the effect of FFF manufacturing parameters on the material properties. Therefore, it is crucial to find and practice the optimal operating conditions best suited for the material. For example, low surface roughness is desired, especially in cases where the components are subjected to cyclic mechanical stress because a smooth surface ensures better fatigue resistance.

### 1.1. Geometry and Time Aspects of FFF Products

In the last years, a considerable number of experiments have been conducted to analyze the impacts of process parameters, like raster angle or layer thickness, on dimensional accuracy, surface roughness and build time. In this section, we will review the state of knowledge in this area to date as well as a short presentation of the latest research conducted on these topics and the research methods used. The thorough analysis of the influence of the process parameters on the mechanical properties as well as the studies on this subject, will be part of the remaining work presented in this paper.

#### 1.1.1. Dimensional Accuracy

Dimensional accuracy—the dimensional agreement between the design specification and the manufactured product—is one of the most fundamental qualities that characterize manufacturing processes. This characteristic allows us to quantify the dimensional error between the design and the product, which is intrinsic to the chosen manufacturing method. Researchers strive to minimize this dimensional error in order to meet the designer’s requirements (usually defined as a “tolerance” or acceptable deviation) and to minimize post-processing. That goal could be achieved by the optimization of certain printing parameters. For example, Akande et al. [5], working on PLA, found that low fill density (the quantity of material inside the part), print speed, and layer thickness (the height of a single layer) fostered the printing of parts with high dimensional accuracy. They also found that high dimensional errors occurred mostly along the thickness (Z direction), suggesting dependence on directionality.

Other researchers like Alafaghani et al. [6] also concluded that thinner layers result in a high level of dimensional accuracy. They further identified the optimal extrusion temperature for dimensional accuracy, suggesting a low extrusion temperature (175–185 °C for PLA). Other studies confirm the trend observed by Alafaghani and Akande regarding the extrusion temperature and the thickness of the layer [7]. The conclusion remains that low extrusion temperature and layer thickness are beneficial for dimensional accuracy.

Researchers such as Nidagundi et al. [8] have extended their research to parameters such as raster orientation and building orientation. An orthogonal Taguchi matrix and S/N ratio were applied for experimental design and determination of optimal parameter levels, respectively. These two parameters, in addition to the layer thickness, were found to have the most impact on dimensional accuracy. The authors conclude that axial printing (0°) on the XY plane produces samples with the best dimensional accuracy.

However, the mechanism of effect in many process parameters, including extrusion temperature, number of shells, infill pattern, and raster width on dimensional accuracy, is substantial and needs further research. Moreover, more than three levels of parameters at the same time should be analyzed to make a more accurate decision and to study the impact of the least known parameters on dimensional accuracy.

#### 1.1.2. Surface Roughness

**Figure 1:**
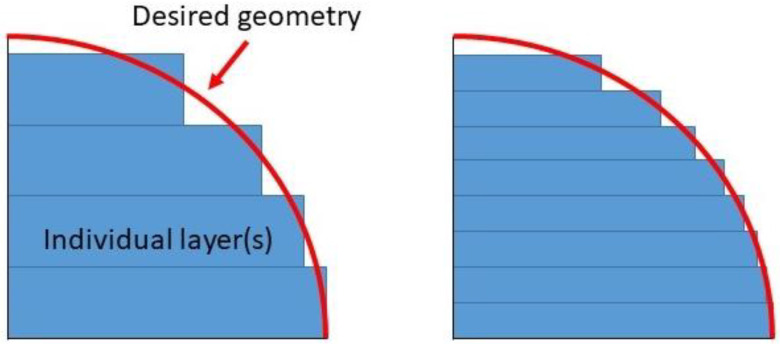
Stair case effect. Note the effect of layer thickness on the structural accuracy and surface roughness.

Surface roughness, defined by the measure of the finely spaced micro-irregularities on the surface texture [9], is a widely used product quality index. In most cases, this characteristic is often used as a technical requirement for mechanical products. The surface roughness impacts aesthetic view and is important to ensure the proper function of very precise parts, such as sealing shafts and friction plates in the automobile sector. One of the limitations of the FFF process is poor surface quality due to the staircase effect (see Figure 1), the resolution of the Standard Triangle Language (STL) file, or directly related to process parameters. The impacts of the staircase effect and STL file resolution depend on the shape complexity of the part and are, therefore, difficult to manage. Nevertheless, a good integration of the process parameters can significantly improve the surface roughness (from 10.7 µm to 2.6 µm Ra by optimization of layer thickness, raster width and print speed) of the desired printed part [10].

In order to measure the impact of layer thickness, build location and orientation of the construction on surface roughness, Wang et al. [11] designed an experiment using the Taguchi method. Their results indicate that the layer thickness was the most significant parameter for the surface roughness. Bakar et al. [12] found similar results that support Wang’s theory. In both studies, it was concluded that a small layer thickness is optimal, as it helps reduce the staircase effect on the printed parts. They, together with Raju et al. [13], also noted that the surface quality of a top surface is better than that of the side surface. Valerga et al. [14] focused instead on the influence of temperature on surface roughness. Analyzing different Polylactic Acid (PLA) filament conditions, they concluded that a low extrusion temperature was preferable for better surface quality.

In conclusion, an improvement in surface roughness can be achieved by selecting a low layer thickness and low extrusion temperature. The surface finish of a top printed surface is better than the side surface, so printing the shorter side of a part in the Z-direction is recommended for the FFF process to reduce the overall surface roughness.

#### 1.1.3. Build Time

Build time is defined as the time that passes from the beginning of a process to its completion. By its very nature, this characteristic depends strongly on the pre-recorded printing parameters, especially infill percent (the percentage of printed material in the volume of the printed part), and can be optimized in the same way as mechanical properties or dimensional accuracy. The goal is thus to obtain the required mechanical and morphological properties of the product in a minimum time.

Nancharaiah et al. [15], as well as Kumar et al. [16] and Rathee et al. [17], investigated the influence of parameters such as layer thickness, air gap (the space between two deposited polymer paths) and raster angle (the angle between the direction of the material deposition and the loading of the part) on build time. They conclude that a high layer thickness and a positive air gap reduce the build time. They also agree that the raster orientation, as well as build orientation (the layup of the printing in the XYZ planes), has a very small influence on the build time.

In conclusion, build time was minimal for high layer thickness, positive air gap and low infill density. Other parameters, such as those related to temperature, need to be further studied and optimized.

### 1.2. Effects of Printing Process on Mechanicals Properties

Depending on the field subject, mechanical properties can be used as one of the guidelines to explore new applications or to determine the expected service life of an existing part. Due to different process parameters (e.g., extrusion temperature and layer thickness), the mechanical properties of an FFF-built part can be different than those of the precursor filament. In the following chapter, current research on the influence of printing parameters on the different mechanical properties, especially tensile strength, compressive strength, and flexural strength, is analyzed and compared in a systematic way. In this section, we will try to explain why the properties of FFF printed parts are inherently weaker than those obtained by conventional methods such as casting and by which mechanisms the printing parameters play a role in the product properties.

Since FFF technology is a method of shaping plastics, it must provide mechanical properties that are at least comparable to other production methods. Many works compare the properties of FFF-printed parts to those obtained by injection molding or compression. S. Ahn et al. [18] tested tension and compression samples made from the same material, ABS P400, which were either injection molded or printed by FFF. The results show that, even with the optimum printing parameters, the tensile strength of the printed specimens is lower (from 5% to 60% less) than that of the molded specimens. Furthermore, Young’s modulus (E) of specimens obtained by printing is between 30% and 45% lower than those obtained by molding.

Moreover, all reviewed studies are based on the proven fact that FFF parts are anisotropic. The developed analytical models for assessing the mechanical properties and the experimental studies are thus designed accordingly [19]. Classical laminate theory is used for describing the orthotropic properties of FFF parts. It should be noted that the anisotropy of printed parts depends not only on the building direction. Manufacturing process parameters such as nozzle diameter, layer thickness, and diameter of the extruded filaments can also significantly affect the anisotropy level of mechanical properties [20].

The study of printing parameters and their influence on mechanical properties is further complicated by the need to identify and characterize the interactions between the different parameters. In FFF technology, a large number of printing parameters can be defined and optimized. The main ones are the raster angle, air gap, and number of shells (number of contours), which will be elaborated in Section 2, as well as the effects of the print direction, print path parameters, the various temperature parameters, and others. All these parameters affect the bonding of the filament and thus influence the physical properties of the final FFF part [21].

**Figure 2:**
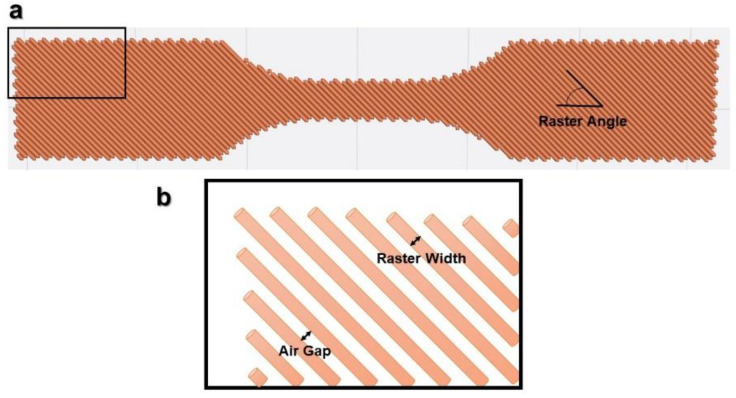
Main print path parameters: (**a**)—print pattern (**b**)—an illustration of a zoom-in on the boxed area.

**Figure 3:**
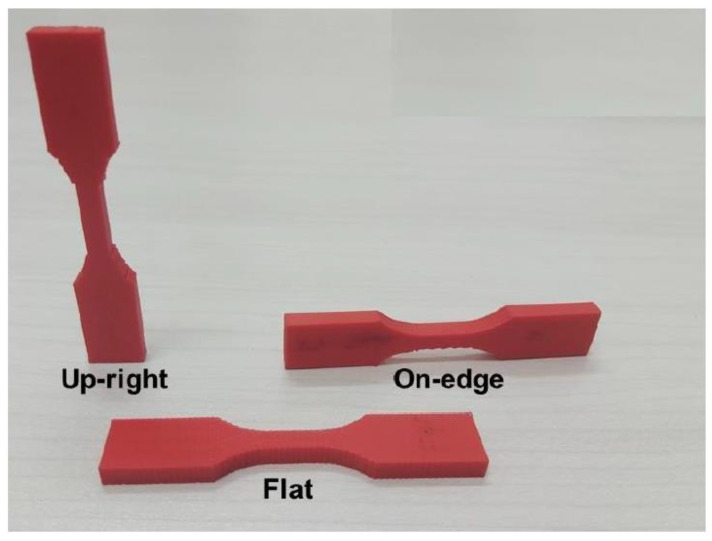
Build orientation.

In Figure 2, the main print path parameters are illustrated. There are other parameter categories, such as environmental parameters (e.g., environment temperature), machine parameters (e.g., print speed) or building parameters (e.g., building orientation, see Figure 3). The raster is defined by the line of molten polymer formed once deposited on the printing structure.

It should be noted that raster angle is sometimes confused with building orientation (Figure 3), probably because the latter can also be described quantitatively (0°, 45°, and 90°). These two parameters and their differences will be developed in Section 2.

As demonstrated by Sood et al. [22], optimizing several printing parameters in order to obtain better mechanical properties does not give the same values of these parameters as when they are optimized independently. In other words, a strong interaction exists between these different parameters. In addition, depending on the type of properties to be improved, the optimal values of each parameter may differ, as is the case for tensile strength and impact resistance [23].

### 1.3. Design of Experiment (DoE)—Evaluating Cross Interference of Printing Parameters

FFF manufacturing is governed by many parameters, some of which are dependent on each other. Most researchers focus on only two to three parameters that they study as variables, while the other parameters are considered constants. The variability of a parameter under study may thus depend on certain constant values assigned to other parameters that are not the subject of the study. In order to obtain a perfect system that provides the absolute optimal combination, it would have been necessary to consider each parameter as a variable. Nonetheless, this approach is very often impractical. A major problem in the study of many variables is the scope of the experiments and their cost, which increases exponentially with each addition of variables. Moreover, the optimal combination of parameters for a given process may be different from the combination obtained through experimental investigation. In order to obtain the optimal combination of parameters despite these constraints, the DoE tool is used by different researchers. In this context, it allows the planning of a series of rigorously organized tests in order to determine, with a minimum of testing and a maximum of precision, the respective influence of the different design or manufacturing parameters of a product in order to optimize its performance and cost.

#### 1.3.1. Taguchi Method

The Taguchi method is one of the most widely used DoE methods, such as by Alafaghani et al. [6], but also Dinwiddie et al. [24], Nidagundi et al. [8], Wang et al. [11], and others. The experimental design proposed by Taguchi involves using orthogonal arrays to organize the parameters affecting the process and the levels at which they should be varies. Instead of having to test all possible combinations as in a basic and systematic approach, the Taguchi method tests pairs of combinations. This method allows for the collection of the necessary data to determine which factors most affect product quality with a minimum amount of experimentation, thus saving time and resources. Taguchi method is best used when there is an intermediate number of variables (3 to 50), few interactions between variables and only a few variables that contribute significantly. Therefore, it is considered a suitable method for the optimization of printing parameters [25]. The parameter design of the Taguchi method includes the following steps: (1) identification of the quality characteristics and selection of the design parameters to be evaluated; (2) determination of the number of levels for the design parameters and possible interactions between the design parameters; (3) selection of the appropriate orthogonal array and assignment of the design parameters to the orthogonal array; (4) performance of the experiments based on the arrangement of the orthogonal array; (5) analysis of the experimental results; (6) selection of the optimal levels of the design parameters; and (7) verification of the optimal design parameters by the confirmation experiment. Therefore, two objectives can be achieved by the parameter design of the Taguchi method: determination of the optimal design parameters for a process or product and estimation of each design parameter’s contribution to the quality characteristics [26].

For example, Alafaghani et al. [6] applied Taguchi’s DoE and an L9 array (which included nine runs with three repeated specimens for each, for a total of 27 specimens) to investigate the main effects of four processing parameters in the FFF process. The processing parameters influence is expressed in terms of the mechanical properties and dimensional accuracy of FFF parts fabricated from PLA. Sood et al. [22] investigated the influence of important process parameters along with their interactions on the dimensional accuracy of ABS. The experimental results indicate that the optimal settings of the factors for each performance characteristic are different, thus mapping the effect of each parameter with respect to the other.

#### 1.3.2. Analysis of Variance

Analysis of variance (ANOVA) is an analysis tool used in statistics that splits an observed aggregate variability found inside a data set into two parts: systematic factors and random factors. The systematic factors have a statistical influence on the given data set, while the random factors do not. Analysts use the ANOVA test to determine the influence that independent variables have on the dependent variable in a regression study. The ANOVA test allows a comparison of more than two groups at the same time to determine whether a relationship exists between them.

(1) $$F=\frac{\mathrm{MST}}{\mathrm{MSE}}$$

where:

F = ANOVA coefficient
MST = Mean sum of squares due to treatment
MSE = Mean sum of squares due to error

The result of the ANOVA formula, the F statistic (also called the F-ratio), allows for the analysis of multiple groups of data to determine the variability between samples and within samples. If no real difference exists between the tested groups, which is called the null hypothesis, the result of the ANOVA’s F-ratio statistic will be close to 1. The distribution of all possible values of the F statistic is the F-distribution. 

There are two main types of ANOVA: one-way and two-way. One-way or two-way analysis refers to the number of independent variables (such as print parameters) in the analysis of the variance test. A one-way ANOVA evaluates the impact of a single factor on a single response variable. It determines whether all samples are identical and is used to determine if there are statistically significant differences between the means of three or more independent (unrelated) groups. Two-way ANOVA is an extension of one-way ANOVA in which there are two independent variables. For example, a two-way ANOVA allows a researcher to compare the tensile strength of a printed material as a function of both extrusion temperature and layer thickness. It is used to observe the interaction between the two factors and test the effect of both at the same time [27].

A two-way ANOVA was used by Ziemian et al. [28] to compare the tension-fatigue properties and the different stress levels associated with the raster orientations tested. These results suggest that the orientation of the ABS filaments relative to the loading axis substantially affects the tension-fatigue properties of the FFF specimens, and the difference between the mean numbers of cycles to failure of each orientation is statistically significant at each stress level. The effect of interaction between stress level and fiber orientation further suggests that the relative influence of fiber orientation is different for each stress level and vice-versa. This conclusion could only be demonstrated by the ANOVA method.

#### 1.3.3. Pareto Optimal Front

**Figure 4:**
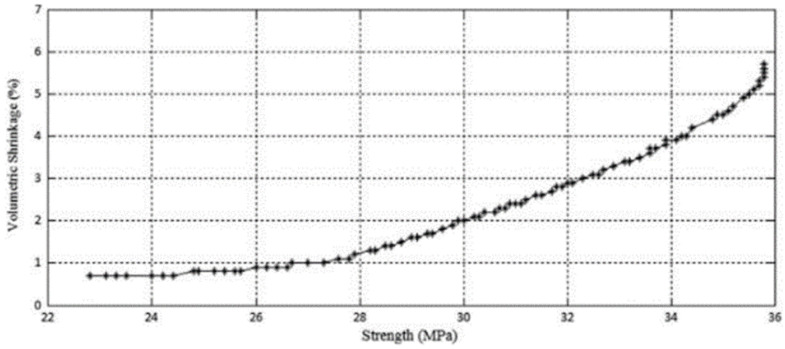
Pareto Optimal Front volumetric shrinkage and tensile strength. The plot visualizes the optimal tradeoff between these two contradictory properties (Reprinted with permission from Ref. [29]. 2014, Taylor & Francis).

Multi-objective optimization problems usually have many optimal solutions, known as Pareto optimal solutions. Each Pareto optimal solution represents a different compromise among design objectives. Hence, the designer is interested in finding many Pareto optimal solutions in order to select a design compromise between contradicting factors that suits their preference structure. There are a number of different methods available for solving multi-objective optimization problems. Gurrala et al. [29], for example, obtained a plot of the response surface which allowed them to describe the relationships between two important quality objectives: strength and volumetric shrinkage of FFF parts and selected process parameters: the build interior, the horizontal build direction and the vertical build. This type of optimization is called multi-objective optimization. It is used when there is an effect of each of the process parameters and their combinations on the two mutually conflicting responses. Resolving these conflicts yields a set of optimal solutions, which is called a Pareto optimal solution, used as a guideline by the designer (see Figure 4).

This type of graph allows the visualization of the relationship between the various optimizations of two different characteristics (here, volumetric shrinkage and tensile strength). The Pareto method helps the designer in quantifying the optimization of one characteristic over the other and in finding the best trade-off between these two properties. For example, in Figure 4, it is easy to see that in order to maximize the tension up to 30 MPa, it will be necessary to accept a volumetric shrinkage of at least 2%.

To conclude, these three methods are the main ones used in the optimization of process parameters. They are the most suitable for this kind of research as they allow for determining the variability and contribution of multiple parameters to the improvement of the quality characteristics. The similarities and differences, as well as the advantages and disadvantages of these methods, can complement each other to best understand and optimize the process to the required results.

## 2. Parameters Optimization

Due to the particularities of additive manufacturing, many process parameters related to additive manufacturing need to be considered while designing parts with an expected functional requirement. The objective of the investigation described in this chapter is to evaluate the effect of each parameter on the 3D-printed components with a focus on the mechanical properties of the product. This evaluation is done by identifying and aggregating a list of pre-published comprehensive, user-controlled process parameters. The effect of these parameters on the mechanical properties, static and dynamics, like Ultimate Tensile Strength, Elastic Modulus and Fatigue Curve (S/N), is then analyzed. We will discover that some of them have a very great influence, like the raster angle or the air gap, while there are certain parameters that influence the mechanical properties only very little or not at all, e.g., the print location on the build platform. The following table (Table 1) summarizes the findings.

### 2.1. Filament Materials

The FFF 3D printing market regularly sees new materials appear: mostly thermoplastic polymers, but also filaments of ceramics and others [4]. In order to FFF print a thermoplastic polymer, a suitable viscosity of the material in the molten state is necessary: sufficiently viscous to provide structural support but fluid enough to allow extrusion. Controlling the viscosity of the melted polymer can be difficult, and the working range can be narrow on some polymers. Moreover, the polymer melting temperature (Tm), together with its glass transition temperature (Tg), dictates the optimal printing temperatures and the temperatures of the platform or printing environment. Furthermore, polymers must necessarily be in the form of filament in order to be extruded [30,31]. In FFF 3D printing, PLA and ABS are historically the two most used polymers, and for a good reason. They have a relatively low melting temperature (Tm), which allows their extrusion at temperatures close to 220 °C, easily achievable by most 3D printers (most printers do not exceed 250 °C, although some specialized ones can reach 500 °C). Over the years, additional thermoplastic polymers have proven to be suitable for this printing process. The currently most used polymers—ABS, PLA and PC—are described below by order of research. Of these three, the main research focus seems to be concentrated on ABS.

#### 2.1.1. ABS—Acrylonitrile Butadiene Styrene

Primarily developed since 1990, ABS is a material made by mixing a styrene-acrylonitrile copolymer with a polybutadiene elastomer material. The flexibility and impact resistant provided by the elastomeric phase, together with the rigidity of the aromatic phase, make it one of the most popular thermoplastics for 3D printing, being ideal for “wear and tear” applications.

ABS requires a hot plate for printing, with recommended nozzle temperatures between 220 °C and 250 °C and bed temperatures around 130 °C. It is generally preferred to PLA when greater temperature resistance is required. 3D printing with ABS allows the creation of parts with precise dimensions, with minimum features as small as 1.2 mm, although it should be noted that the printer parameters and the complexity of the part mainly determine the level of precision [31,32]. ABS also lends itself well to various post-processing techniques like sanding, painting, gluing, milling, drilling, and cutting.

In addition, ABS can be used to create materials with improved properties such as biocompatibility, conductivity and translucency. It can also be mixed with other materials to achieve superior mechanical properties, such as PC-ABS (polycarbonate-ABS), which has superior strength and heat resistance [32,33].

#### 2.1.2. PLA—Polylactic Acid

PLA is one of the widely used thermoplastics in FFF. It is usually derived from corn starch, making it a biodegradable, moisture-sensitive polymer. Its biodegradability by hydrolysis makes it a “green polymer”, and so its use has increased in the last years [34,35,36].

PLA needs relatively low energy and temperature to process prototypes and functional parts with good quality due to its low glass transition temperature of 50 °C. These days, many desktop 3D printers use PLA as a filament as it does not require a heated bed, although it is prone to jamming a printer nozzle during printing due to its tendency to absorb moisture. PLA has higher tensile strength and lower warp but lower ductility than ABS. The temperatures recommended for printing are about 210 °C for the nozzle and 70 °C for the bed [19].

PLA’s main advantages are its low printing temperature and ease of printing. Compared to other thermoplastics, PLA has a relatively low printing temperature (~80 °C, compared to 250 °C for ABS). Therefore, PLA is less likely to warp and clog the nozzle during the printing process, pending low enough humidity. Also, compared to ABS and other thermoplastics with higher melting temperatures, PLA typically produces better surface details and sharper features. Furthermore, PLA is one of the easiest material filaments to 3D print, with the material easily adhering to a variety of surfaces and doesn’t require a heated print bed, adding to its ease of use. Finally, PLA is an eco-friendly material, being biodegradable and non-toxic. Compared to petroleum-based thermoplastics, which take thousands of years to break down, PLA parts are typically compostable within a few years or even months. On the other hand, PLA has a low heat resistance and, therefore, cannot be used for high-temperature applications. In high temperatures, PLA can rapidly deform, especially under loads. PLA is typically weaker and has a lower tensile strength than its counterparts, PET-G (not to mention high-performance polymers such as PEEK). Since PLA parts, when 3D printed, are quite brittle, the material is more suited for aesthetic purposes rather than mechanical [32].

#### 2.1.3. PC—Polycarbonate

Polycarbonate (PC) is among the most widely used industrial thermoplastics. It has exceptional qualities in terms of thermal resistance and impact resistance (approximately double that of ABS). This is a highly strong, resistant thermoplastic that’s often used for making compact discs, bullet-proof glass, and other products where durability is a key factor. It has a high impact strength and a transparent look that’s highly attractive to many users. As with ABS, though, it requires ventilation during printing, as it produces a lot of fine particles that can irritate users’ eyes and clog printer heads if they are not properly maintained. It is also more prone to warping than other materials.

PC is one of the strongest engineering plastics available for 3D printing. The mechanical properties of the PC make it the ideal material for demanding technical environments or applications requiring high levels of flexural strength and tensile strength. PC can be quite challenging to 3D print since its glass transition temperature is relatively high (~161 °C), which makes it difficult to print on domestic printers. Furthermore, PC has a greater tendency to warp and split than other thermoplastics such as ABS. However, once mastered, it can produce strong and durable 3D-printed parts for engineering applications [32].

### 2.2. Path Parameters

The path parameters are the parameters that govern the configuration and method of depositing the molten polymer on the printing platform. These parameters allow varying the characteristics of the raster (deposited polymer path), such as its height, width, distance from the previous deposit and angle of deposition (with respect to the loading direction). The path parameters are among the most influential on the mechanical properties. In addition, the path parameters drastically influence other aspects of the printing process, such as the printing time, surface roughness, and dimensional accuracy.

#### 2.2.1. Raster Angle

Raster angle, or raster orientation, is defined by the direction of the material deposited (roads) relative to the loading of the part [18]. This parameter is among the most influential on the mechanical properties and thus is thoroughly investigated.

**Figure 5:**
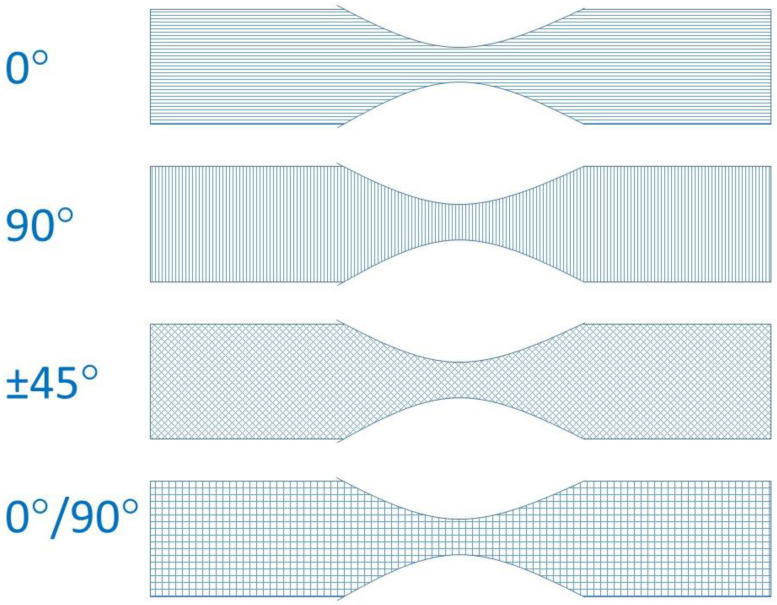
Various common raster angles.

Raster angle can vary continuously (depending on software increments) from 0° to 179°, but the main angles used are shown above (Figure 5), though some researchers have investigated less common angles such as 15/75° or 30/60° [26,27]. The influence of this parameter on the tensile strength of ABS was first studied in 1996, when the FFF was not yet widespread [37], and was followed by more comprehensive studies [18,28,38]. All concluded that the tensile strength was maximum when the raster angle was 0°. The small raster angle means that the rasters are inclined in the direction of the load, and the breaking strength becomes less dependent on the weaker inter-fiber bond for axial rasters than for transverse rasters. These raster angles will therefore offer more tensile strength. This trend regarding the influence of the raster angle on the tensile strength is confirmed both for ABS [39,40,41,42] and for PLA [43]. The ratio of the highest strength (0° orientation) to the lowest strength (90° orientation) is about a factor of two. For example, Rajpurohit et al. [43] found that it was possible to improve the tensile strength of PLA from 24 to 44 MPa simply by varying the raster angle. The effect of raster orientation is not limited to tensile strength: this raster angle also optimizes dimensional accuracy, surface roughness and build time [8].

Nonetheless, some researchers conclude differently, such as Dawoud et al. [44], whose experimental results showed that the tensile and impact strength was maximum when the raster angle was 45°/−45°. The average tensile strength values for the 45° weft angle in another case [30] were 20.13 MPa versus 18.36 MPa for 0°, a difference too small to reach any conclusion. A recent study [45] highlighted the effects of raster angle by choosing to compare 0/90° and 45/−45° and noted an unexpected difference between the two polymers tested: While the PLA specimens reinforced the main trend regarding the influence of raster angle on tensile strength with a maximum when the raster angle was 0/90°, the ABS specimens show a higher UTS for a raster angle of 45/−45° than for 0/90°. For ABS, the tensile strength averages vary by 1.8 MPa, or 6.5%, between 45/−45° and 0/90° in favor of the 45/−45° raster angle, while for PLA, the tensile strength averages vary by 2.6 MPa, or 5%, between 45/−45° and 0/90° in favor of the 0/90° raster angle (10 specimens for each variables combination). These variations are too small, probably less than the standard deviation that was not reported, to establish a definite trend or an interaction with the filament material. Moreover, this research focused only on configurations combining two angles (0/90 or 45/−45) without comparing them to standard configurations like 0° or 90°. However, it is still possible to affirm that these researches are in the same direction as the ones exposed at the beginning of the sub-chapter. In fact, the unanimous tendency admits 0° as the optimal value for the raster angle and the decrease of the tensile strength proportionally to the increase of the raster angle. Therefore, better proprieties are obtained with an angle of 45° than with an angle of 90°. For combined angles, the 45/−45° configuration might produce better performance than 0/90° because the 90° layer weakens the structure more than the 0° layer improves it.

There is only one study comparing 0° and 0/90° [18], but it is possible, from the previously cited research, to predict the trend of the results. We can assume that since the axial rasters resist the stress better than the transverse rasters, unidirectional 0° specimens should be stronger than 0/90° cross-plies—in the primary axis. The main reason for this phenomenon is the fact that resistance to fracture becomes more dependent on the weaker inter-fiber bonding of the transverse rasters, as well as the presence of porosity at the interface.

**Figure 6:**
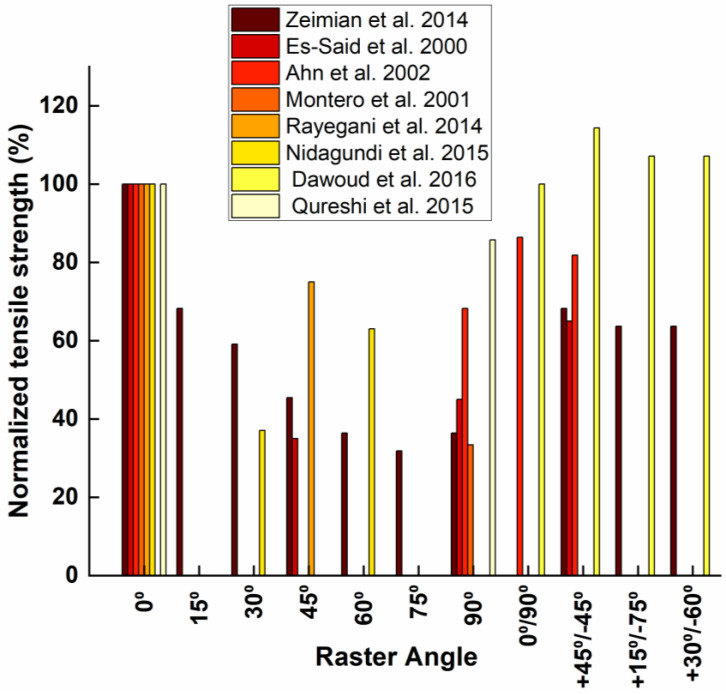
Summary diagram of the different research on the raster angle. For comparison, results were normalized to the 0° UTS or, if none are presented, to the 0/90° [8,18,28,30,38,40,42,44].

To summarize the effect of raster angle (see Figure 6), these results clearly show that the layer orientation of the rapid-prototyped samples affects the tensile strength and confirm the fact that FFF parts are anisotropic as mentioned above—in a somewhat similar way to continuous-fiber laminated composites. The 0° orientation, also called axial orientation, which has the characteristics of coinciding the direction of the raster with the loading of the part, is usually the optimal orientation to obtain the maximum tensile strength.

#### 2.2.2. Air Gap

**Figure 7:**
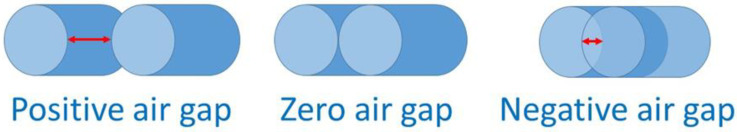
Air gaps.

The air gap, also named raster to raster gap, is a configurable parameter in most systems and is defined by the space between two adjacent rasters of FFF material (see Figure 7). In lots of works (featured below), it is categorized as a parameter with significant influence on the mechanical properties of the product. The default air gap is often set to zero, meaning that the rasters just touch. It can be modified to leave a positive gap, which means that the rasters of material do not touch, or a negative gap, meaning that two rasters partially occupy the same space. The positive gap results in a loosely packed structure that builds rapidly. The negative gap results in a dense structure, which requires a longer build time [38]. The range in which the studied values (gap) usually vary is −0.05 mm to +0.05 mm.

The first studies about the influence of the air gap on the tensile strength appeared in 2001 [18,38,44] and concluded that the tensile strength was maximum when the air gap was as negative as possible. In an attempt to quantify the optimization potential of this parameter, Rayegani et al. [42] studied the influence of this parameter over the range from −0.00254 mm up to +0.022 mm and found that it was possible to improve the tensile strength of the polymer from 6.14 to 34.07 MPa (for ABS specimens), approximately a 6-fold increase, simply by varying the air gap. In order to explain this seemingly unanimous trend, Mohamed et al. [46] studied six of the major process parameters, including the air gap, and concluded that with decreasing air gap, all mechanical performance properties increased significantly. The variation in mechanical properties as a function of the air gap can be explained by the fact that a zero or negative air gap produces very close rasters with reduced porosity. Another possible explanation can be the improvement of cohesion due to the overlapping of the rasters. This proximity of the rasters leads to a dense structure and stronger cohesive force between the interlayers or across the filaments, and thus improves the raster dynamic mechanical properties, but also results in poor dimensional accuracy [46]. A zero air gap improves diffusion between adjacent rasters but can also decrease heat transfer as well as total bonding area [47]. It should be noted while analyzing these trends that the eventual air gap in the printed part might be different than the one set due to the effects of viscosity and thermal expansion of the printed material during the printing process.

Although the trend of air gap influence on the mechanical properties of the polymer seems monotonous, air gap also interacts with other parameters to change their trend of variation. For example, the variation of the air gap from positive to negative value can inverse the tendency of some parameters like the raster width and the raster angle. Montero et al. [38] found that when the tensile specimen has its rasters oriented in the transverse direction, a negative air gap will increase the performance. Then again, when the rasters are oriented axially, the air gap effect is less prominent, although the same trend is still observed.

**Figure 8:**
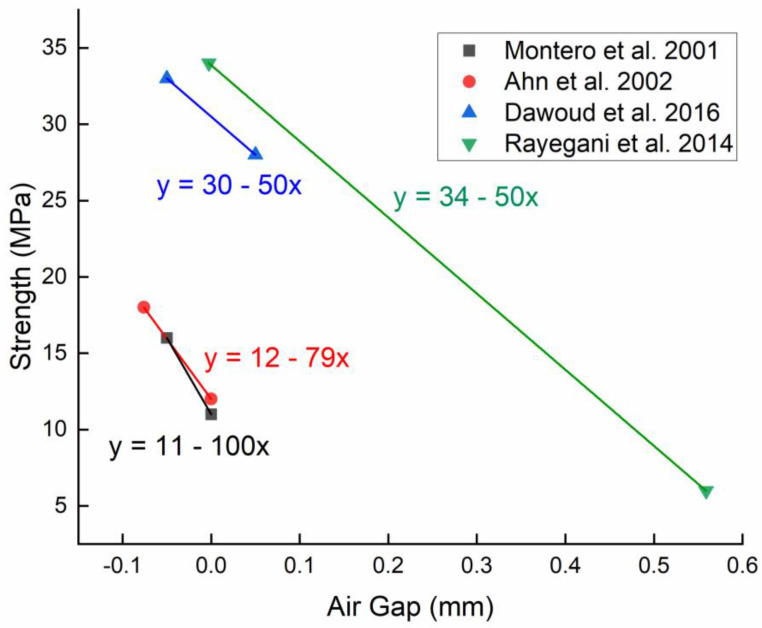
Summary diagram of the air gap effect on printed ABS tensile strength. It can be seen that the tensile strength is maximal when the air gap is as negative as possible [18,38,42,44].

The air gap emerges as a dominant parameter in the optimization of mechanical properties. It seems clear (see Figure 8) that tensile strength was maximal when the air gap was as negative as possible. Nevertheless, such an increase comes at the expense of heat dissipation between the rasters and thus might negate the dimensional accuracy due to residual stress formation.

#### 2.2.3. Layer Thickness

**Figure 9:**
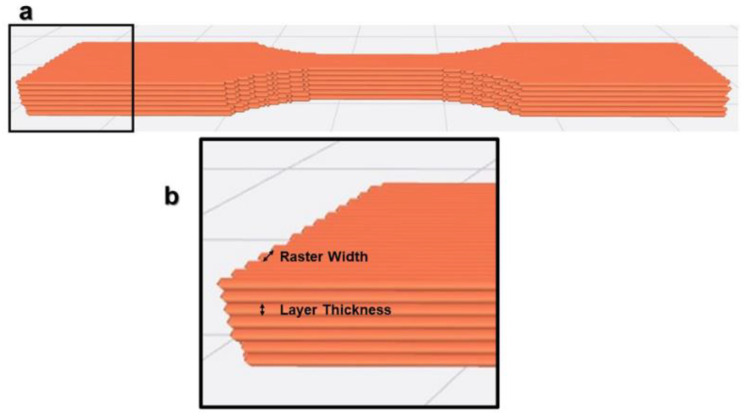
Raster width and Layer thickness of 3D printed dog-bone ((**a**)—full scale. (**b**)—close-up).

The layer thickness, which corresponds to the height of a single layer (see Figure 9), depends on the diameter of the extruder nozzle and is about 25–75% of it (inversely proportional to the raster width). Due to the high disparity of the results, a clear trend of influence on mechanical properties is hard to identify, but it is obvious that layer thickness has a certain influence on mechanical properties.

Layer thickness has been studied by Tymrak et al. [45] as a parameter with a high potential to influence mechanical properties. The highest tensile strength they found (60.4 MPa, measured on PLA) was obtained at 0.2 mm layer thickness, then dropped by 20% at around 0.3 mm (to 48.5 MPa) and ended with a more modest 13% improvement at 0.4 mm (54.9 MPa). It should be noted that the standard deviation is unusually omitted from the report, making it impossible to assess the significance of the variations observed. They obtained the same trend for ABS but with layer thickness having smaller effects. A similar trend was observed by Nidagundi et al. [8]. This trend resembles a V-shaped figure in which the minimum tensile strength would be obtained in the middle of the studied range. A close (but not identical) trend was observed by Sood et al. [23], in which the tensile strength first decreases very slightly and then increases as the thickness of the layer increases. They explained this observation by the stress accumulated across the raster’s width. However, the same stress accumulation also increases temperature near the bodied surfaces, which can improve diffusion and result in the formation of a strong bond and, thus, better mechanical properties. Another group [46] concluded that by increasing the layer thickness, a smaller number of layers were required. Fewer layers, they claim, reduced the residual stress and deformation of the part and improved its strength. Rankouhi et al. [48] investigated only two different levels of this parameter and thus obtained a linear and decreasing graphical representation. It seems that by extending the range of layer thickness values, they would have obtained a tendency similar to Tymrak, Nidagundi or Sood (V-shaped).

Regarding PLA, one study [43] showed that the tensile strength decreases with increasing layer thickness. Higher tensile strength was observed at the minimum layer thickness. This can be explained by the fact that thinner layers have a relatively larger inter-layer contact area (with respect to the raster volume), leading to higher bonding strength. Higher stiffness is also obtained at a lower layer thickness for the same reason, leading to a better load transfer. Similarly, low stiffness and low strain at failure have been observed with thicker layers, and the presence of voids and the reduced contact area between the wefts leads to a brittle failure mechanism.

A fundamental difference is observed between XY (flat) and ZX (upright) orientations regarding the influence of layer thickness on mechanical properties. At the XY orientation, a higher layer thickness results in higher tensile strength, as it increases the contact length between neighboring wefts. At the ZX orientation, a lower layer thickness was preferred as the increased compression between rasters promotes larger contact areas [49].

**Figure 10:**
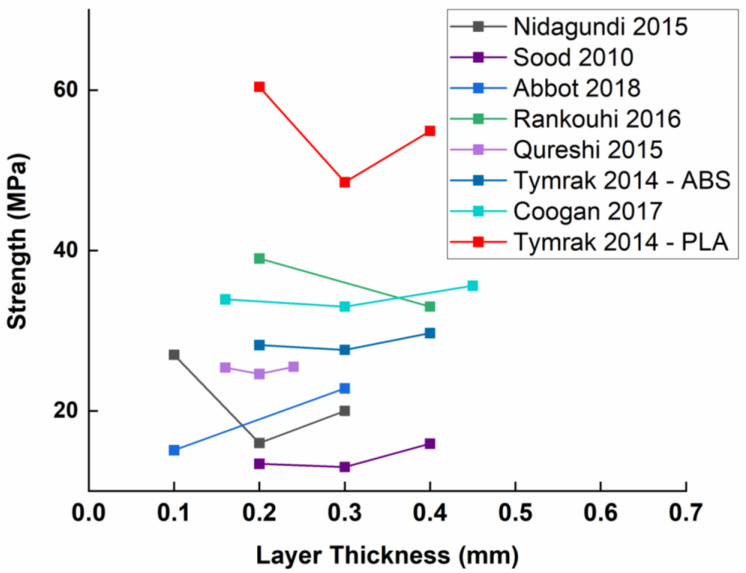
Summary diagram of the different research on the layer thickness (Blue lines—ABS, Red line—PLA). The large variety in absolute values observed is most likely due to very different printing conditions between the research groups [4,8,23,30,45,48,49].

The thickness of a printed layer appears to be a dominant parameter in the optimization of mechanical properties (see Figure 10). Nevertheless, paradoxically, there is no consensus about the trend of this influence. We, therefore, conclude that layer thickness is a parameter with a high level of interaction with several other parameters, and these interactions must be isolated in order to establish a trend. Moreover, one could possibly identify a kind of V-trend, suggesting competing mechanisms affecting raster-to-raster adhesion.

#### 2.2.4. Raster Width

A raster width is the width of the deposition path related to tip size (as shown in Figure 9) and depends mostly on the extrusion nozzle diameter. The raster width is usually studied between 0.2 mm to 0.7 mm. A study encompassing five process parameters (air gap, raster width, extrusion temperature, filament color, and raster orientation) designed the experiment on ABS polymer using a fractional factorial design [38]. The experimental results showed that although air gap and raster orientation had a vast influence on tensile strength, raster width had very little influence on the mechanical properties of the printed material. Panda et al. [50] maintained this negligible effect of raster width on the tensile, flexural, and impact strength of the test sample. Similar results were found by Sood et al. [47], who, based on the experimental results, established that all the parameters studied, except the raster width, were significant.

However, other studies [42] have found that the tensile strength was greatest at the minimum raster width (measuring between 0.2032–0.5588 mm), at about a 13% difference between the two extremes. Furthermore, examining the effect of raster angle, layer height, and raster width on the tensile properties of the FFF-printed PLA, Rajpurohit et al. [43] found that raster width and its interaction with layer thickness have the potential to improve the tensile properties. Using fractography with a high-precision measuring microscope, they found that increasing the raster width from 0.5 mm to 0.6 mm increased the tensile strength from 44.5 ± 2.26 MPa up to 47.3 ± 2.69 MPa, but then decreased to 45.6 ± 3.04 MPa as the raster width is increased to 0.7 mm. As the standard deviation is higher than the variations found, the effect seems, again, negligible. However, the contact area between adjacent layers at 0.6 mm width is larger compared to that at 0.7 mm-raster width, where the contact area between the adjacent layers is lesser, and the presence of voids can also be seen at the high raster width. The presence of voids can act as a stress concentrator between layers since cracks may be easily initiated and propagated, ultimately resulting in lower tensile strength at 0.7 mm raster width. Due to the presence of voids, the effective cross-section area has also been reduced and can also be the reason for reduced tensile strength at 0.7 mm raster width. Similarly, Liu et al. [51] found that the maximum value of the raster width (0.5 mm) is the optimal width to maximize the tensile strength of printed PLA parts.

**Figure 11:**
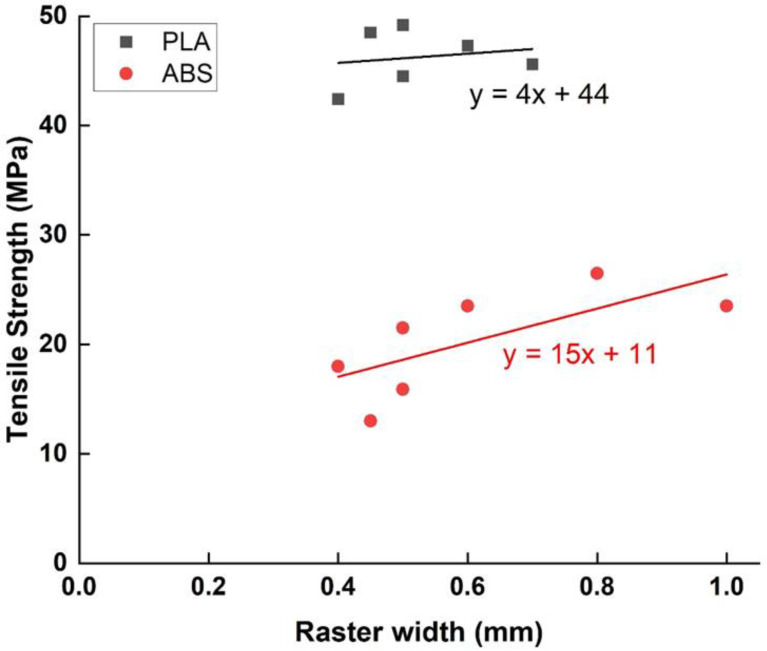
Summary diagram of the raster width effect on tensile strength of ABS [4,38,47] and PLA [43,51].

The influence of raster width on mechanical properties is usually too small to be identified in a single study. However, a macro approach of all studies, which covers a range of almost one millimeter (Figure 11), shows a small but clear improvement of the mechanical properties with increasing raster width. This trend is explained by the fact that an increase in raster width leads to a decrease in the number of rasters per unit area. The density and homogeneity of the piece thus increase, and the strength of the structure relies on fewer raster/raster cohesive bonds—which are usually the weakest link. It can also be seen that the effect of width on ABS is much higher than on PLA. This difference can be attributed to the much higher thermal conductivity of ABS (0.171 W/m·K° on average for ABS vs. 0.0439 W/m·K° for PLA [52]), allowing a more efficient evacuation of the heat produced by the printing process.

### 2.3. Building Parameters

The building parameters are the parameters that govern the configuration and the method of model construction in the space. They allow the variation of the printed object characteristics such as its orientation, density, internal structure and number of contours. The building parameters (a.k.a. construction parameters) are among the most influential on the mechanical properties. Moreover, they have the particularity to have a direct impact on the success of the printing process and a poor control of these parameters risks not only the mechanical and dimensional properties of the product but also the success of the printing process itself.

#### 2.3.1. Building Orientation

The building orientation (see Figure 3) consists of three possible printing directions, named according to the axis coinciding with the length of the sample, but other orientations can also be used. The first experimental study about the impact of build orientation on the tensile properties of polycarbonate suggested that the flat build orientation was appropriate for optimizing tensile strength, followed by the On-Edge orientation [53]. It should be noted that printed parts in this study showed a decrease in modulus of approximately 45% compared to the raw material, as well as a decrease in final tensile strength of 30–60% compared to the raw material. The influence of build orientation on the mechanical properties of ABS explored using the Taguchi method and verified by Gray’s theory and Technique for Order Preference by Similarity to Ideal Solution (TOPSIS), established that the tensile strength of the FFF part was significantly higher when the test samples were printed on the edge deposition orientation. In contrast, the worst tensile strength was observed when the test samples were in the direction perpendicular to the layer (Z or upright) [11]. Others, such as Cantrell et al. [54], presented results for both ABS and polycarbonate in different build orientations (flat, edge and upright) and showed that the tensile strength was highest when the build orientation was on edge for PC specimens and flat for ABS specimens. No rational explanation was found for the difference between the two polymers, so the author justifies this result by the difference in the printers used (PC was printed in a Fortus 360mc™ which is of better quality than the one used for ABS—Ultimaker^®^ 2). Other studies of the build orientation effect on the ultimate tensile strength property of ABS concluded that the 0° orientation (flat or face up) produced not only maximal tensile strength but also optimized dimensional accuracy, surface roughness and fabrication time [8,41]. Furthermore, build orientation was found to be more significant than raster orientation, and a close relationship between surface roughness and mechanical properties was observed.

**Figure 12:**
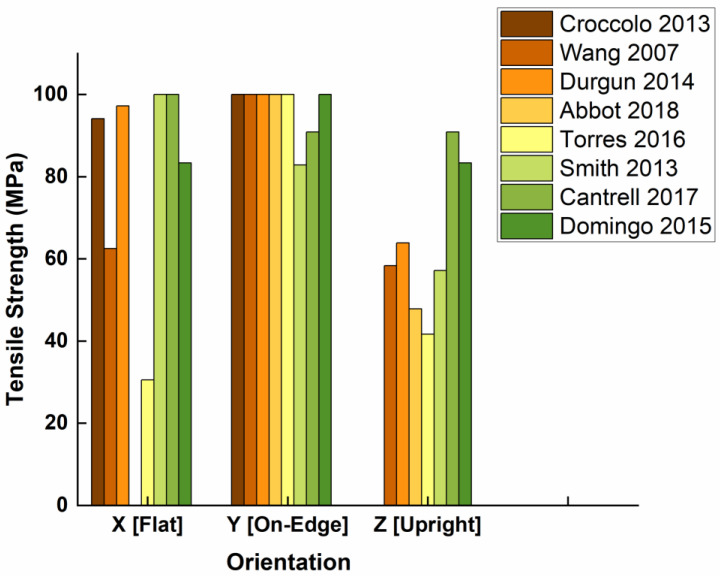
Summary diagram of the different research on the build orientation. Note that the results are always normalized to the strongest building orientation [11,20,41,49,53,54,55,56].

In the graph below (Figure 12), the data have been grouped by paper and normalized to the maximal tensile strength in order to neutralize the influence of external parameters such as the quality of the material or the precision of the printer. From the body of research cited above, it can be concluded that orientation is an important and influential parameter on mechanical properties, and in particular on tensile strength. It has been established that the tensile strength is highest at either a flat or on-edge build orientation. In these orientations, the contact area with the platform is the largest and therefore allows for better temperature uniformity throughout the printing process. The orientation with the lowest performance is the Upright orientation. As can be seen in Figure 12, the Upright orientation will almost always be weaker than either of the other two (Flat and On-Edge). In this orientation, breaking is caused by an “adhesive” failure, where the contact points between layers are broken, whereas in the Flat (and On-Edge) orientations, breaking the sample requires breaking solid lines of extruded polymer.

#### 2.3.2. Number of Shells

**Figure 13:**
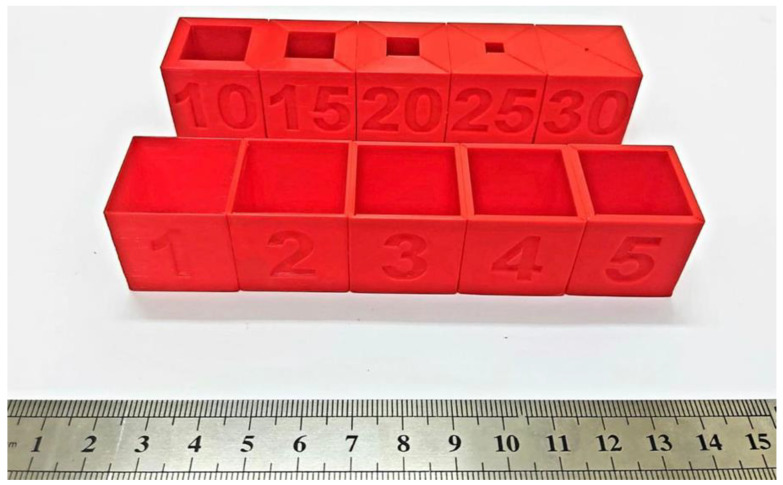
Number of shells.

The number of shells or contours (Figure 13) is defined by the number of rasters to build around all outer and inner part curves. Additional contours may improve perimeter part walls. This parameter is among the more influential on the mechanical properties.

The first work demonstrating a correlation between the number of shells and mechanical properties was carried out by Mahmood et al. [57]. They found that the ultimate tensile strength (UTS) is inversely proportional to the cross-sectional area of a specimen while being directly proportional to the number of shells, all other parameters being constant. An analytical model was developed in another study by Croccolo et al. [55] to predict the strength and stiffness properties as a function of the number of contours deposited around the edge of the component (a.k.a. “shells”) and the setting of other key parameters of the deposition process. They found that the tensile strength was maximum at the maximum number of shells (in a range of one to 10 shells). Other works studying fewer shells (one to three) confirm this trend [30,46]. A statistical analysis of variance (ANOVA) indicates that layer thickness, air gap, and the number of shells have the most impact on the fatigue properties, optimizing for maximum fatigue at the maximum number of shells (10) and minimum road width (0.4572 mm).

**Figure 14:**
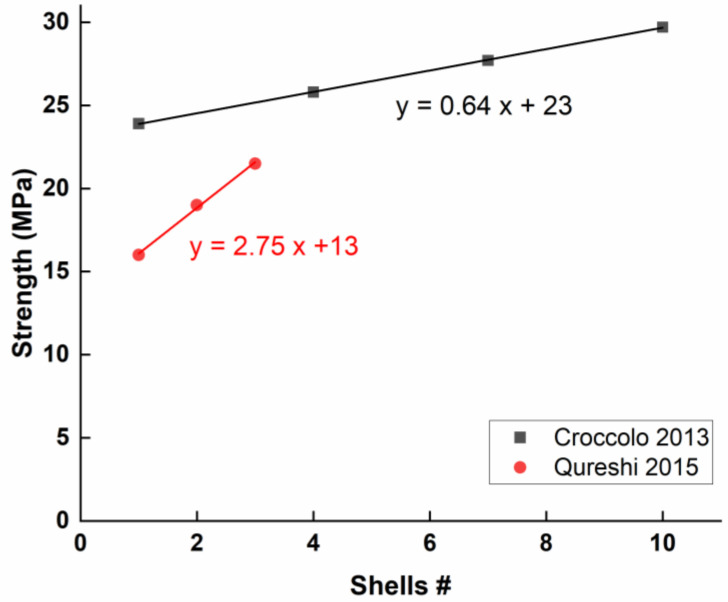
Summary diagram of the different research on the number of shells. Two of the studies cited above did not reveal enough data to allow a graphic representation [30,55].

All the research that has investigated this parameter agrees that increasing the number of contours results in an increase in the mechanical properties in general and the tensile strength specifically (Figure 14). Nonetheless, during the design of a part intended for printing, the increase in the number of contours is mainly confronted with constraints such as the printing time, the cost of the raw material, and the volumetric weight of the printed part, which increases rapidly with each added contour.

#### 2.3.3. Infill Density and Pattern

**Figure 15:**
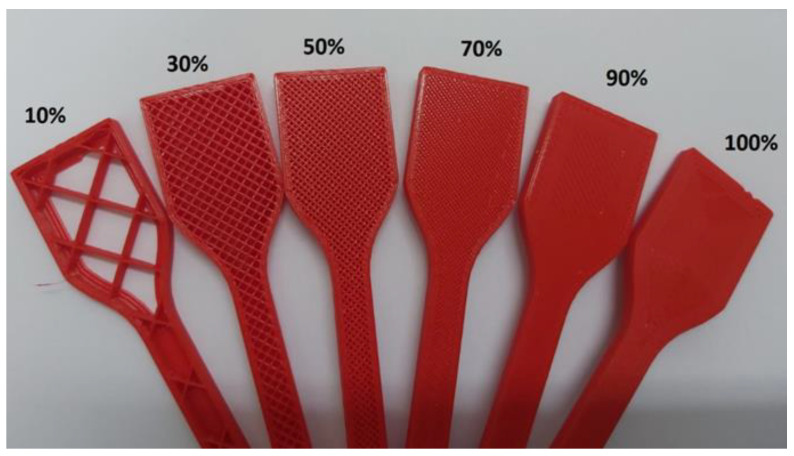
Infill density.

The internal structure, commonly known as the infill, is an invisible inner part covered by the outer layer(s) and can have different shapes, sizes, and patterns. Infill density or infill percent (Figure 15) is defined as the relative quantity of material inside the part. The greater the percentage of fill, the better the mechanical properties of the part, with the downside of longer printing time and the amount of material to be used [58].

**Figure 16:**
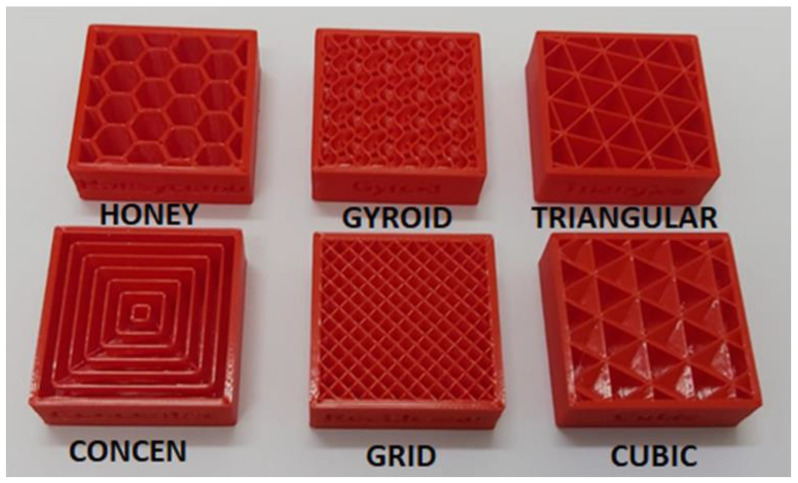
Different infill patterns used in FFF printing. Note the high variability of the geometry and symmetry groups.

Moreover, different infill patterns are used in parts to produce a strong and durable internal structure. Hexagonal (“honey-comb”), diamond (“cubic”), and linear (“grid”) are commonly used infill patterns (Figure 16).

The infill density is a parameter with a critical influence on most mechanical properties. Torres et al. [56] studied the influence of infill density on the mechanical properties of FFF samples made with PLA and found that it is possible to improve the tensile strength significantly by increasing the infill density (from 17 MPa with 35% infill to 27 MPa with 100% infill). Typically, infill density is the most influential or second most influential parameter (after layer thickness for modulus of toughness and fracture energy) in ranking printing parameters by influence on mechanical properties. However, regardless of the optimization carried out, it is clearly established in this research that the mechanical properties of PLA improve directly and consistently with the increase in infill density, whether it be stiffness, strength or ductility. The same authors, in another research [59], found that the combination of 100% infill with a low thickness of 0.1 mm is favorable for obtaining maximum values for all the properties of the material. Kim et al. [60] have confirmed this trend, showing that the 100% filled sample had a higher tensile strength (up to 60% improvement) than the 50% filled sample.

It should be noted that according to the simple rule of mixtures, an increase in density of X% should lead to an increase in tensile strength of X%. However, we observe that the rule of the mixture does not apply to the infill percentage, suggesting that other mechanisms are in play here. Lužanin et al. [61], in an experimental study, confirm this influence and find a significant interaction between raster angle and infill density, as well as a non-linearity of effects. They demonstrate that a maximum bending strength greater than 116 N can be achieved with both factors at their central level. The contour plot also shows that the 10% infill can also produce a higher force when combined with a high level of frame angle (60°), thus significantly reducing the total build time while maintaining the bending force at 10% of the maximum. However, when interpreting these findings, the inherent porosity of printed parts, even at 100% infill, should also be accounted for.

The specific effect of the infill pattern was studied by Alafaghani et al. [6,62] in a range of density values from 20 to 100%. They found that the infill pattern was less influential on the mechanical properties than building orientation, extrusion temperature and layer height for samples with a high infill percentage (above 80%). In another study [30], experimental results demonstrated little or no effect of the infill pattern on the tensile strength of printed parts in a much lower infill percentage (8% infill percent). However, we believe that to highlight the potential influence of infill patterns, samples with lower infill percentages should be further studied to provide bigger room for infill patterns.

**Figure 17:**
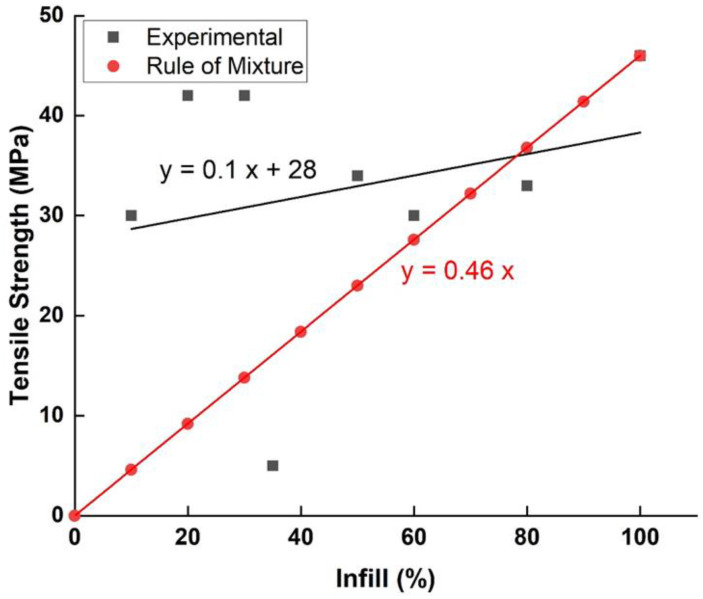
Summary diagram of the different research on the infill percent [58,61,62]. Rule-of-mixture presumes voids have zero strength.

The precise nature of the infill percent influence on mechanical properties and its ranking among the most influential parameters is far from being agreed upon by all. Current research, however, as well as general knowledge, allow us to conclude that the tensile strength is maximal when the infill density is high. Nevertheless, this relationship does not follow the rule of mixtures that predicts the mechanical properties of composite materials, as can be seen in Figure 17. To learn more about the influence of the infill percent on the mechanical properties, it is important to study in depth the possible interactions between the infill percent and other main influencing parameters. Regarding the infill pattern, research agrees that it has little influence on the tensile strength at high infill density. Its influence combined with low infill density sample, on the other hand, still needs to be explored and could produce surprising and important results.

### 2.4. Temperature Parameters

Despite vast interest in the optimization of the FFF processing parameters, there are relatively few works focusing on the overall effect of temperature parameters, and specifically of the extrusion temperature, on the mechanical properties. Nevertheless, this parameter is significant and must be studied and monitored in order to control the printing process and optimize mechanical properties.

In the 3D-printing process, the part is built one layer at a time. Each layer is deposited onto the previous layer, which can be referred to as the substrate layer. The first layer is deposited onto a build plate or build platform. In some cases, this build plate may be heated to reduce residual stresses in the part. In FFF, the thermoplastic material, typically in a filament or pellet form, is fed into a heated extruder, where it is heated beyond the melting temperature. The viscous material is then extruded through a small orifice onto the heated substrate or substrate layer. While hot and viscous, the material can bond with the previous layer, but once it has cooled down below its glass transition temperature (Tg), this bonding mechanism no longer takes place. Therefore, the longer the material is kept above its Tg, the better the bond between layers. The z-strength (strength in the direction perpendicular to the layer plane) is thus expected to be improved if the previous layer is above the Tg during the deposition of the new layer. In contrast, if the temperature of the thermoplastic is too high and the viscosity too low, the part may distort or collapse under its own weight [6].

#### 2.4.1. Nozzle Temperature

The nozzle or extrusion temperature is defined by the temperature at which the filament is heated during the deposition process of molten polymer on the printing platform. The extrusion temperature depends on various technical parameters related to the printing process: the type of material, the printing speed, and, most importantly, the viscosity of the polymer at the melting temperature. The nozzle temperature had a significant effect on layer-to-layer bond strength, as it governs the initial temperature of the fiber being deposited [24]. This temperature thus has a direct effect on the temperature of the interface, which governs the reptation of the polymer molecules (thermal motion of very long linear, entangled macromolecules in polymer melts or concentrated polymer solutions) and, therefore, the diffusion and void-healing across the polymer layers.

**Figure 18:**
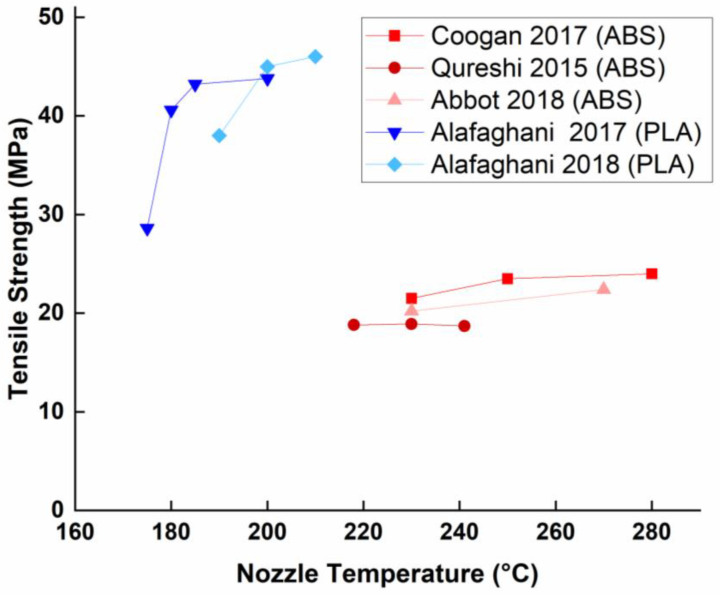
Summary diagram of the different research on the extrusion temperature—Red: ABS studies [4,30,49]—Blue: PLA studies [6,62]. The lines are only to guide the eye and ease the identification of trends and different data-sets.

As seen from Table 2 and Figure 18, ABS is not sensitive to extrusion temperature variations in the area in which it is (physically) printable. It is, therefore, difficult to obtain a statistically measurable optimization. The first real mechanical study was performed by Coogan et al. [4] and revealed that the upper temperature limit of a nozzle could reach 280 °C (Tm + 80 °C). However, the difference in performance between the two temperatures is almost negligible (33.8 MPa for 230 °C versus 34.4 MPa for 280 °C, less than 2%). Another study [30] reinforces this hypothesis in the 218–241 °C range by studying the influence of extruder temperature on the mechanical properties of ABS. They concluded that the influence of temperature is insignificant to the performance of the material. Other works [18,49] have extended the range of extruder temperature to 230–270 °C and again concluded that the extruder temperature played a more minor role than the other parameters studied (printing speed and orientation).

Unlike ABS, PLA does react to temperature changes in the nozzle. Increasing the temperature results in a decrease in viscosity, which improves coagulation, entanglement, and, therefore, the strength of the bonds between the wefts. One study [62] proves this hypothesis by examining the effects of extrusion temperature on the mechanical properties of PLA over the range of 175 to 205 °C. The results show that increasing the temperature from 175 °C to 205 °C improves the tensile strength by almost 53% (from 28.6 to 43.8 MPa) and Young’s modulus by 54% (from 1947 to 3004 MPa). Although this increase is considerable, it should be remembered that there is still a non-negligible difference compared to the properties of the raw material (43.8 to 59 MPa for tensile strength, respectively). Torres et al. [58] confirmed this trend at even higher temperatures (215 °C–230 °C) and again found the temperature of 230 °C (Tm + 80 °C) optimal for all tested loading situations (tensile and fracture). If the PLA is heated beyond that point, the too-low viscosity might clog the extruder or lead to structural deformation, affecting the physical and aesthetic properties of the printed component. In a more recent study on the influence of FDM processing parameters on the dimensional accuracy and mechanical properties of PLA specimens by Taguchi DOE L9, Alafaghani et al. [6] demonstrated that increasing the extrusion temperature leads to an increase in the stiffness of the specimens (from 38 to 46 MPa) due to a greater cohesion between layers, but only up to 200 °C. The experimental results show a significantly increased stiffness when the temperature elevated from 190 °C to 200 °C but negligible one in the range between 200–210 °C. Dinwiddie et al. [24] also conclude that increasing the extrusion temperature reduces the viscosity of the filament and thus increases its cohesion.

Intuitively, the nozzle temperature seems to be a significant parameter of the mechanical properties of the print. The expected trend is that the higher the temperature of the extruder, the better the mechanical properties of the product. However, this logic seems to apply only to PLA printing, as ABS does not seem to react to variations in extrusion temperature, and most of the research that has analyzed the influence of extrusion temperature on ABS has classified it as a non-significant parameter. This difference in behavior can be explained by the graphical representation of the relationship between polymer viscosity and temperature.

**Figure 19:**
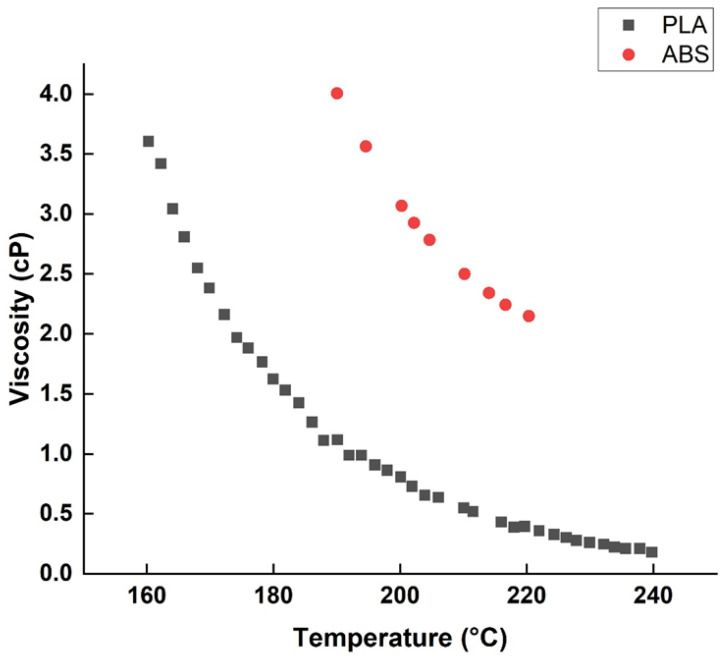
Diagram of viscosity vs. temperature of ABS & PLA [63,64].

The graph (Figure 19) shows that the variability of PLA viscosity is significantly higher than that of ABS. For PLA, a reduction in viscosity from 3.5 to 2 cP only requires a drop of 12 °C, whereas ABS requires a drop of more than 25 °C for the same reduction in viscosity. Also explained by the viscosity is the fact that PLA is affected differently by variations in extruder temperature, depending on the range tested. According to various research, it seems that above 200 °C, the variations become much less important, if not insignificant [6]. In addition, it seems that the optimum extrusion temperature for printing ABS is around 260 °C while that of PLA is around 210 °C. Based on the respective glass transition temperatures (Tg) of the two polymers, we can conclude that the optimal extrusion temperature is approximately equal to Tg + 150 °C.

#### 2.4.2. Platform Temperature

**Figure 20:**
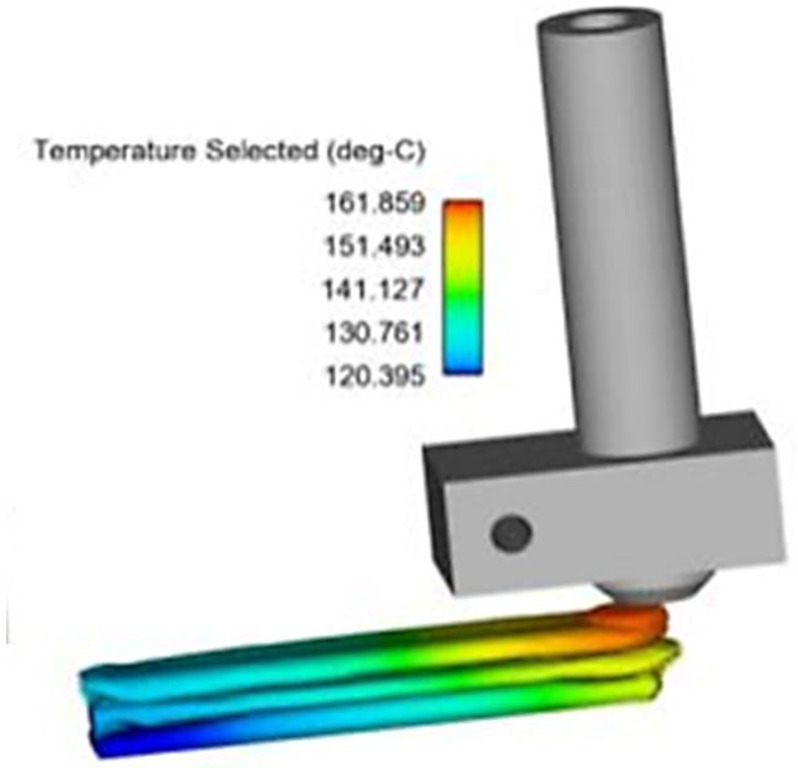
Thermal distribution of multi-layer PLA printing process [65]. © 2023 TechConnect http://techconnect.org. Reprinted and revised, with permission, from the Informatics, Electronics and Microsystems: TechConnect Briefs 2018, pp. 118–121, 13 May 2018, Buffalo, NY, USA.

Polymers, by their nature, have shrinkage and expansion properties under the effect of temperature. For most polymers (except Reverse Thermal Gelation polymers), the hotter the environment to which it is exposed, the more it will expand. Conversely, as the material cools, it will shrink on itself. Therefore, in most printing systems, the first layer is not cooled by the fan blowing on the print at the nozzle but rather remains hot thanks to the heating plate or an adhesive surface, maintaining the first layer in a slightly expanded state. However, if the platform temperature is higher than Tg + 40 °C (e.g., 150 °C for ABS), the excessive expansion will result in poor dimensional accuracy. The ideal temperature is thus around the polymer glass transition temperature (Tg). Heating the platform also reduces the temperature gradient between the new hot layers and the old cooled layers throughout the printing process (see Figure 20), thus preventing the deformation of the printed structure.

**Figure 21:**
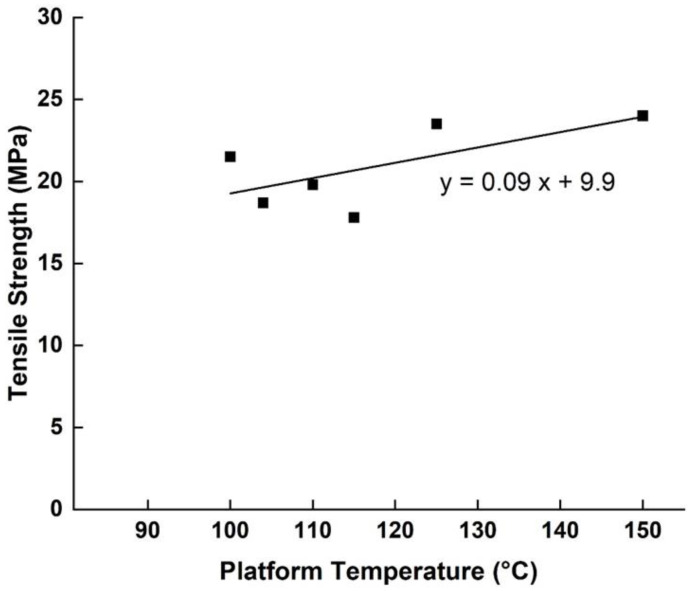
Summary diagram of the different studies on platform temperature. The negligible slope suggests little influence of platform temperature on the tensile strength [4,30].

Coogan et al. [4] studied the effect of platform temperature between 100–150 °C on mechanical properties. They found that platform temperature was the only parameter that did not have a statistically significant effect on bond strength. Nevertheless, it is understood that while variation in this parameter does not affect the final mechanical properties (as demonstrated in Figure 21), the platform temperature is important to ensure the adhesion of the first layers to the platform and, thus, the success of the printing process (preventing the construction from collapsing during the printing process). To confirm this hypothesis, it would have been necessary to count the number of samples disqualified for geometrical deformation as well as the number of aborted printing cycles.

The optimization of the platform temperature remains a very poorly studied subject. However, it can already be concluded that it is not a parameter influencing the mechanical properties directly in a major way. Nevertheless, it does remain a determining factor in the outcome of printing at the structural and dimensional levels.

#### 2.4.3. Thermal Effects and Environmental Temperatures

The thermal effects occurring during the printing process were also studied, as they influence the surface quality, the dimensional accuracy and the mechanical properties of the parts. The contribution of the various heat transfers occurs through three mechanisms: convection, conduction and radiation. If the printing environment is heated, the convection transfers between this environment and the deposited filaments appear to be the most important. Another major source of contribution is the conductive exchanges between the filaments and the platform on the one hand and between the filaments themselves on the other hand. These convective and conductive exchanges should be considered when modeling the FFF process [66]. Investigating the temperature profiles of the extruded filament by heating the printing environment to 50 °C, 60 °C, and 70 °C demonstrated that the hotter the environment, the higher the minimum profile temperature. In other words, the deposited filaments stay longer at a temperature higher than their Tg and are heated up to it more easily during the deposition of the neighboring filaments. This maintenance at a higher temperature favors the diffusion phenomena between filaments and thus improves their bonding strength. On the other hand, the fracture profiles obtained showed differences depending on the location of the part on the bed. This can be explained by a heterogeneous air flow in the printing space, resulting in different convection conditions [67].

Precise control of the cooling conditions after extrusion is the key to obtaining better properties and quality of the printed parts. A slow cooling process prevents a too-steep temperature gradient that would cause distortions within the layers or between the layers, a distortion that results in lower mechanical properties and dimensional accuracy. Faes et al. [68] demonstrated this theory by studying, without heat input, the influence of the inter-layer time (time between two consecutive layers) on the mechanical properties. They found that the longer the inter-layer time, the lower the tensile strength. Therefore, it is important to keep the deposited material at a temperature as close as possible to the extrusion temperature for as long as possible. 

**Figure 22:**
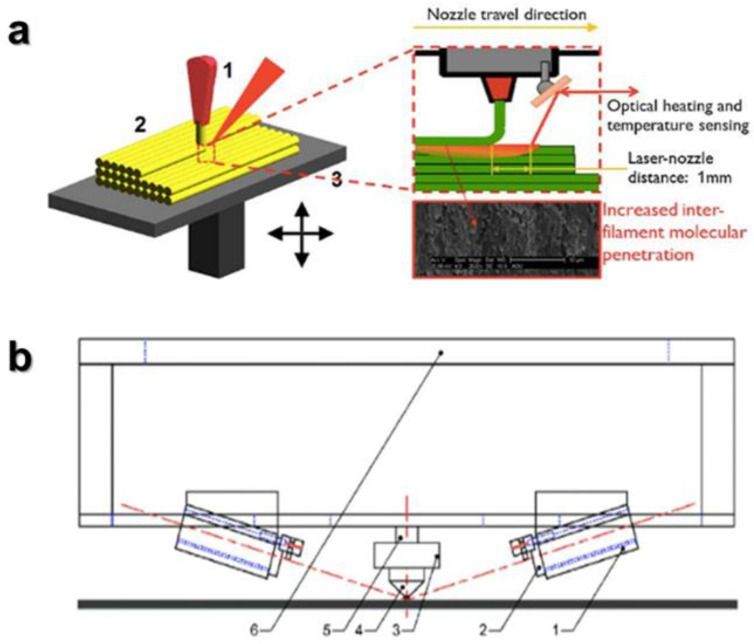
Concept illustrations of the improved FDM process with laser-assisted heating. (**a**) pre-deposition heating approach, where a laser beam heats up the substrate locally where the new interlayer interfaces are forming. In doing so, higher interface temperatures are obtained, which allows more polymer inter-diffusion across the interface to increase FFF part strength in the build direction. Reprinted with permission from Ref. [69]. 2016, Elsevier. (**b**) Schematic diagram of the improved FDM process with a laser-assisted heating device. Here the laser is heating the extruded filament. (1) fixed device, (2) infrared fiber laser, (3) heater, (4) nozzle, (5) liquefier, and (6) support. Reprinted with permission from Ref. [70]. 2019, Elsevier.

The addition of local laser heating near the nozzle during the deposition of the extrudate has also been investigated by two groups of researchers. Ravi et al. [69] chose a positioned laser that heats the filament of the lower substrate layer just before the deposition of a new filament on top (Figure 22a). This procedure resulted in a 50% increase in interlayer bonding strength and a more ductile interlayer failure mode, with a visible presence of plastic deformation zones. A clear increase in flexural strength was thus observed, from 33 to 49 MPa obtained with a laser of 1 Watt (optimized in the 0.2–2.2 Watt range).

On the other hand, two configurations of the position of two lasers (Figure 22b) were studied by Du et al. [70] a lateral configuration, heating the extruded filament before deposition on each side, and a pre- and post-deposition configuration, where the two lasers are placed along an axis parallel to the printing path. The most efficient configuration was the lateral, allowing better cohesion between layers, an improvement in precision and a 195% increase in the tensile strength of the printed part.

Due to the nature of the technology, temperature parameters play an important role in obtaining the desired mechanical properties. Among the parameters developed in this section, the temperature of the extruder remains the most influential parameter having a direct effect on the viscosity and deposition of the polymer and an indirect one on all aspects of the cooling process.

## 3. Parameters Interactions and Interrelations

The in-depth study of the influence of printing parameters on physical and mechanical properties also includes knowing and studying the possible interactions that certain parameters have on others. In fact, it is possible to identify trend changes in certain parameters due to variations of one or more other parameters in the printing process. In order to control the properties of the result, it is thus important to consider the main interactions (briefly discussed in the previous chapter) that exist between the printing parameters.

Rayegani et al. [42], using a GMDH model to investigate the influence of four printing parameters on the tensile strength of printed ABS parts, found that independently of raster angle and part orientation, there is an interaction between raster width and air gap. When the air gap is negative, the mechanical properties improve as the raster width decreases. Inversely, with a positive air gap, the raster width must be increased to improve mechanical properties.

**Figure 23:**
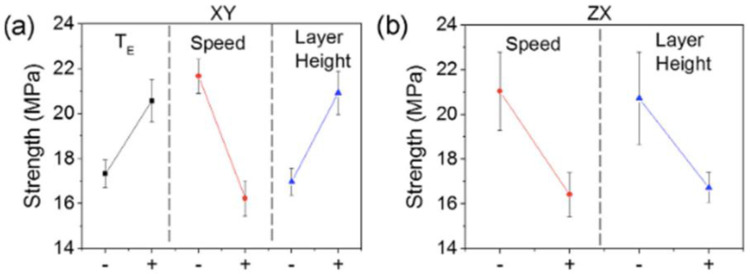
Main effects plots of tensile strength as a function of print parameters in (**a**) XY and (**b**) ZX orientations. Reprinted with permission from Ref. [49]. 2018, Elsevier.

The magnitude of the air gap effect on the mechanical properties is further influenced by the raster angle [38]. When the tensile specimen has its rasters oriented in the transverse direction (90°), the variation of the air gap towards negative values has a great influence on the tensile strength, up to a 600% difference (from 2 MPa to 12 MPa). On the other hand, when the rasters are oriented axially (0°), the variation of the air gap has an almost negligible effect on the tensile strength—less than 10% (from 20 MPa to 22 MPa). The orientation of the building can also interact with other parameters, such as layer thickness, to reverse the trend of effect: at XY (flat) orientation, the layer thickness should be increased to obtain better mechanical properties (from 17 to 21 MPa), but at ZX (upright) orientation, increasing the layer thickness results in poorer mechanical properties (from 21 to 16.8 MPa), as can be seen in Figure 23 [49].

Investigating three process parameters, Rajpurohit et al. [43] found a strong interaction between layer height and raster width but no interaction between other factor combinations. For a raster width lower or equal to 500 µm, the tensile strength is proportional to the layer thickness. But for a raster width greater than 600 µm, the trend is reversed, and the best mechanical properties are obtained at the minimum of the layer height. A 5 process parameters study [71] has demonstrated a positive correlation between nozzle diameter and layer thickness, showing the tensile strength of PLA samples achieved at the maximum layer thickness (raster width was not mentioned). This study also mentioned the previously unspecified “extrusion velocity” (the material feeding speed through the extruder) and “filling velocity” (the moving speed of the printer head). However, too few studies were found on these parameters to be accounted for.

By considering the polymer selection as a parameter, we tried to find interactions between the type of polymer and other parameters such as raster angle or build orientation. As demonstrated above through several studies investigating the influence of extrusion temperature on mechanical properties, the mechanical properties improve with increasing the extruder temperature [6,59,62], but only when printing PLA. ABS does not seem to react to variations in extrusion temperature, and most of the research which analyzed the influence of extrusion temperature on ABS classified it as an insignificant parameter [4,18,38,49].

Another difference, this time between PC and ABS, was highlighted by Cantrell et al. [54]. Through an in-depth study of the influence of build orientation and raster angle on mechanical properties, a clear distinction of the influence of these parameters between ABS and PC can be seen. For both materials, it can be stated that the optimal orientation is the flat orientation (XY); however, for ABS, the optimal raster angle is 0/90°, while for PC, the best properties are obtained with an angle of +45/−45°. This inversion of tendency between the raster angles +45/−45° and 0/90°, which occurs only for the flat orientation, is specific to ABS. A similar difference was noted by Tymrak et al. [45] comparing ABS to PLA. It can be observed in this study that the layer thickness has a similar influence on ABS and PLA, while the raster angle shows a trend that is reversed between the two polymers. One should therefore use a raster angle of 0/90 for PLA and 45/−45 for ABS to obtain the optimal mechanical properties. However, this interaction should be taken with caution because the variations found are too small, probably less than the standard deviation that was not reported. Rodriguez–Panes et al. [72] have also found a difference in variability by studying ABS and PLA: this time in the filling percentage, the layer height and the raster angle. The results obtained with ABS show a lower variability of the mechanical properties according to the variations of these parameters than PLA (12% for ABS vs. 20% for PLA). Regarding Polycarbonate, there are not yet enough studies available to assess the overall variability of the polymer compared to ABS or PLA.

Assessing trends in the effects of printing parameters on mechanical properties is therefore dependent not only on the pre-set, untested parameters but also on the printed material itself. These interactions, together with the growing interest in “open code” printers, make a comparative study both highly needed and highly complicated.

## 4. Future Trends

FFF technology can easily benefit from recent advances in the world of artificial intelligence in general and machine learning in particular. In fact, the process of printing parameters optimization can be carried out by an algorithm similar to computer simulations predicting tensile strength while reducing the waste of raw material. In this way, it is possible to predict the quality of the parts as well as the potential defects and imperfections that may be created during printing. In the future, Machine Learning (ML), which is defined as computer programming to optimize a performance criterion based on example data or past experiences, can be a disruptive technology. For machine learning in 3D printing, besides the typical application of making predictions through data fitting, the research community is exploring new and innovative approaches to integrate ML and Artificial Intelligence (AI) methods into printing. ML algorithms, applications, and platforms are helping 3D printing practitioners improve product quality, optimize manufacturing processes, and reduce costs. These applications span printing parameter optimization, mechanical and electrical property prediction, defect detection, geometric deviation control, and quality prediction and assessment. Research efforts in ML focus on new materials analyses and the optimization of manufacturing plans as well as an automated in-process feedback system for AM, which will help advance smart 3D printing in the near future [73].

The ability to print new polymers, which are far more resilient to ambient conditions, is also a potential avenue for advancing FFF technology into mass use. High-performance polymers are described as high-temperature thermoplastics that can be used at ≥150 °C based on values of heat distortion temperature and continuous use temperature. High-performance thermoplastics (PEEK in particular) are almost as strong as aluminum, reaching tensile strengths of up to 100 MPa, while being generally 50% lighter, making them very popular materials in the manufacturing industry. Polyaryletherketones (PAEK), which include Polyetherketone (PEK), Polyethyletherketone (PEEK) and Polyetheretherketoneketone (PEKK), are an important family of high-performance thermoplastics. In addition to their excellent thermomechanical properties and good chemical resistance, they offer the advantage of not being hygroscopic, i.e., they do not absorb moisture when exposed to it. These polymers can be obtained by heating from their glassy state or by slow cooling from their molten state. M.Vaezi and Y. Shoufeng [74], in 2015, reported the successful printing of PEEK structures by extrusion. Two systems were tested, one using heated PEEK powder extruded through a syringe, and the other, using the more familiar fused filament deposition (FFF) process. It was found that proper management of the heating of the material in the extruder head is necessary to avoid its degradation and to control its viscosity during extrusion. This control is not possible with the syringe extrusion process, as non-uniformity of the printed layers and excessive viscosity due to overheating have been observed. On the other hand, it was possible to install a heat sink in the heating zone of the extrusion head on the FFF process. Using an extrusion temperature between 410 and 430 °C, a printing platform at 130 °C, and heating the printing environment with lamps to approximately 80 °C, the printing was successful. Wu et al. [75] also experimented with PEEK 3D printing. The results prove that the deformation is minimal with a chamber temperature of 130 °C and a nozzle temperature of 350 °C. The PEEK parts were successfully printed using the optimal parameters determined by the experiment. The results of this study further demonstrate the feasibility of manufacturing customized PEEK functional parts in small batches in the industrial and medical fields. But the use of high-performance thermoplastics is still not without its flaws. For example, as mentioned earlier, the post-processing steps are still complex. Other approaches focus on even more innovative materials that meet the specific requirements of each industry. In the long term, one might wonder whether high-performance materials might not compete with the development of metal 3D printing.

In the same spirit, composite materials are also a development vector for 3D printing. Many research projects are being conducted regarding printing carbon-reinforced polymers in FFF, which is an emerging manufacturing technology for high-performance applications. The intrinsically limited strength and dimension stability of the fabricated FFF plastics pushed toward developing fiber-reinforced polymer (FRP) 3D printed components via FFF. Additions of various reinforcement kinds have been investigated to improve the static mechanical properties: glass fibers [76,77,78], carbon fibers [79,80], or powders [81,82] (e.g., metals or metal-oxides). Fibers can be short or continuous [77,83,84,85,86]. Concerning the thermoplastic used, most researchers have used ABS in the form of resin, granules or filament. PLA and Polypropylene (PP) [77] were also investigated as a matrix.

Last, but not least, the possibility to 3D print materials that can respond to stimuli (a.k.a. “4D printing”) is one of the more exciting developments toward “materials as devices”. Such materials can be photoresponsive [87], thermoresponsive (directly [88,89], or indirectly using magnetic or resistive heating [90]). Their vast applications, from soft robotics through smart textiles to the aerospace field, make them highly sought-after.

## 5. Conclusions

This work reviews a large number of printing parameters and their potential influence on the characteristics of the parts produced by the FFF printing process. These characteristics are many and diverse; thus, the present study focuses mainly on the mechanical properties produced by FFF from the three most common polymers. The number, as well as the variability, of the parameters, have led existing research to address the optimization of the FFF process parameters using different tools such as DoE (Taguchi methods and others), statistical tools (ANOVA) and other optimization methods. A range of significant parameters to ensure the control and repeatability of the characteristics obtained by 3D printing emerged from this study. Furthermore, we have defined and described the interdependencies between these parameters through diagrams in order to get an overview of what ensures the quality of the printed parts and the efficiency of the FFF process.

FFF technology has as many parameters as the creativity and ingenuity of printer designers allow. Over the years, printers have become increasingly sophisticated and therefore allow a multitude of printing parameters to be varied as the user wishes. However, not all of them have the same influence on product characteristics: raster angle, air gap, build orientation, and the number of shells are the four most influential parameters on the physical and mechanical properties, regardless of the material used. Hence they have become, over time, the most studied parameters. Layer thickness also appears to be one of the main parameters of FFF printing. However, the nature of its influence on mechanical properties is far from unanimous and strongly interdependent with the aforementioned parameters. It is thus difficult to say what strategy to adopt for this parameter without resorting to experimental tests.

Among the printing parameters, the temperature parameters also have significant effects. In particular, the extrusion nozzle temperature and the printing platform temperature must be adapted to the material used, as they have a direct impact on the quality of the printed part. Nozzle temperature is one of the parameters with the greatest effect on the quality and strength of the printed object, as the viscosity of the extruded material depends directly on it. Increasing the environmental temperature or the addition of a local material heating system in the FFF process improves not only the surface quality but also the bonding strength between layers, as it encourages diffusion across the interfaces. Unfortunately, the temperature parameters remain much too little studied, probably because of the expensive and complex experimental material they require to be studied.

Some “rules-of-thumb” can be derived from this meta-analysis. For example, in all the materials examined, a negative air gap maximizes mechanical properties at the expense of dimensional accuracy, a raster angle of 0° maximizes tensile strength and minimizes printing time, and the number of shells maximizes all mechanical properties, both static and dynamic, but increases the manufacturing time.

The potential of this technology remains largely unexplored, and there are several approaches that need to be addressed: the in-depth study of the characteristics of PC, which is very little studied in the context of FFF printing despite great advantages like its tensile strength; the investigation of two or more polymers printing using the same system as well as the same initial conditions; the in-depth study of the temperature parameters influence on the mechanical & dimensional properties; the ability of the platform temperature to increase or decrease the printing process success; and to deepen the interactions between the different parameters discovered in this study. This data infrastructure will be necessary for new research groups to explore and deepen these untapped vectors, such as composite materials and machine learning, in order to extend the applications of FFF in many other industry sectors.

## Figures and Tables

**Table 1:** Classification of printing parameters by their influence on the main mechanical properties. Different properties are marked with different color. Color gradient indicates the strength of the parameter effect on the property.

Influence	Tensile Strength	Flexural Strength	Compressive Strength
*Critical*	Filament material	Filament material	Filament material
*Considerable*	Raster Angle	Build Orientation	Layer Thickness
*Highly*	Air Gap	Raster Angle	Build Orientation
*Greatly*	Infill %	Layer Thickness/Infill%	Raster width/Infill%
*Quite*	Number of Shell	Air Gap	Air Gap/Infill Pattern
*Little*	Raster width	Raster width	Extrusion Temperature
*Insignificant*	Environment temperature	Print Speed	Environment temperature

**Table 2:** Thermal properties of the polymers studied and mapping of extruder temperature studies.

Material	Authors	Tested Range (°C)	Significant
ABS Tg: 110 °C Tm: 200 °C	Ahn et al. [18]	270–280	no
	Montero et al. [38]	270–280	no
	Coogan et al. [4]	230–280	no
	Qureshi et al. [30]	218–241	no
	Abbott et al. [49]	230–270	no
PLA Tg: 60 °C Tm: 150–160 °C	Alafgani et al. [62]	175–205	yes
	Torres et al. [58]	190–210	yes
	Alafgani et al. [6]	215–230	yes
PC Tg: 147 °C Tm: 265 °C	-	-	-

## Data Availability

No new data were created in this study.
